# Potential value of three-dimensional ultrasonography in diagnosis of diabetic nephropathy in Chinese diabetic population with kidney injury

**DOI:** 10.1186/s12882-020-01902-w

**Published:** 2020-06-29

**Authors:** Nan Li, Yi-ru Wang, Xiao-qi Tian, Lin Lin, Shu-yuan Liang, Qiu-yang Li, Xiang Fei, Jie Tang, Yu-kun Luo

**Affiliations:** 1 Medical School of Chinese PLA, Beijing, 100853 China; 2 Department of Ultrasound, The First Medical Center, Chinese PLA General Hospital, Beijing, 100853 China

**Keywords:** Diabetic nephropathy, Type 2 diabetes, Non-diabetic renal diseases, Three-dimensional ultrasonography, Contrast-enhanced ultrasound

## Abstract

**Background:**

To explore the potential value of three-dimensional ultrasonography (3DUS) and contrast-enhanced ultrasound (CEUS) in the diagnosis of diabetic nephropathy (DN) in Chinese diabetic patients with kidney injury.

**Methods:**

Patients with type 2 diabetes mellitus and kidney injury in our hospital were enrolled, and the clinical characteristics as well as the laboratory data of patients were collected; 3DUS and CEUS were used to evaluate the morphological structure and blood perfusion of kidneys. Eligible patients were categorized into two groups based on renal biopsy results: DN group and non-diabetic renal diseases (NDRD) group. Correlation analysis and logistic regression analysis were applied to identify the risk factors of DN development.

**Results:**

A total of 115 patients aged from 24 to 78 years old were recruited in the experiment, of which 64 patients (55.65%) and 51 patients (44.35%) were in the DN group and NDRD group, respectively. After correction to CKD stage, BMI and right kidney volume index were retained to identify patients with DN. The ROC of the logistic regression model had an AUC of 0.703 (95% CI: 0.591–0.815) and it was higher than both indicators.

**Conclusion:**

3DUS has potential value in the diagnosis of diabetic nephropathy in Chinese diabetic population with kidney injury and may act as an auxiliary diagnosis for DN. More prospective and well-designed studies with larger samples are needed to verify the result.

## Background

Diabetes mellitus is one of the most common metabolic diseases in the world. It is estimated that the overall prevalence rate of adult diabetes has reached 10.9% in 2013 in China [1]. Diabetic nephropathy (DN) is one of the most common and important complications of diabetes. The pathological process of renal changes is quite diverse, which can be observed from early hyperfiltration with an increased glomerular filtration rate (GFR) to late nephrosclerosis and fibrosis. An estimated 30–40% of type 2 diabetes mellitus (T2DM) patients are afflicted by nephropathy [2]. Nondiabetic renal disease (NDRD) refers to diabetes with other kidney diseases, such as IgA nephropathy (IgAN) or membranous nephropathy (MN).

The pathogenesis, clinical manifestations, treatment, and prognosis are different between DN and NDRD. In addition, NDRD has a relatively better prognosis since it is usually treatable and even curable, whereas the renal lesions in DN are believed to be more irreversible [3]. Renal biopsy is considered as the canonical standard to discriminate DN from NDRD [4]. However, renal biopsy is an invasive test that is associated with several risks. In practice, a large proportion of patients with type 2 diabetes mellitus (DM) are not formally evaluated by renal biopsy [5, 6]. Instead of pathological diagnosis, DN patients are usually diagnosed based on their clinical symptoms. In that case, NDRD patients are likely misdiagnosed as DN and thus could not be properly treated. Therefore, it is very important to discover a new non-invasive diagnostic indicator for the clinical diagnoses of DN and NDRD.

Clinical practice guidelines for the disease management of type 2 diabetes recommend the screening for microalbuminuria [7]. However, some patients with T2DM with estimated GFR less than 60 ml/min per 1.73 m^2^ did not present microalbuminuria [8]. Hence, two-dimensional ultrasound is not sensitive enough in detecting early renal changes like renal size changes or parenchymal flow variation.

Three-dimensional ultrasonography (3DUS) was invented in the early 1990s. In 3DUS, volume datasets of echo information from a large series of two-dimensional ultrasonographic images are quickly acquired and assembled by a computer [9]. Contrast-enhanced ultrasound (CEUS) has significant advantages over CT and blood test due to the absence of ionizing radiations, and the lack of the risk of nephrotoxicity [10]. The emergence of CEUS represents a significant breakthrough in imaging, as it is an effective, repeatable, noninvasive, and economic imaging technique [11]. Both of these two newly developed ultrasonic techniques could be used to identify DN from NDRD. Herein, the aim of our study is to explore the potential value of 3DUS and CEUS in the early diagnosis of DN.

## Methods

From April 2017 to November 2018, a case control study was conducted in our hospital. Demographics and clinical data (medical history, laboratory index and ultrasonic data) were collected from electronic medical record (EMR) after patients were admitted to the hospital before having renal biopsy. This study was approved by the Medical Ethics Committee of Chinese PLA General Hospital (No. S2017–152-02), Beijing, China.

### Population

Inclusion criteria: (1) Both male and female patients aged between 18 to 80 years old; (2) Clinically diagnosed as type 2 diabetes; (3) Renal damage (the presence of proteinuria); (4) Definite renal pathologic findings; (5) Inpatients in the department of nephrology in our hospital.

The pathological types of NDRD include: membranous nephropathy, IgA nephropathy, obesity-associated nephropathy, focal glomerulosclerosis, hypertensive renal damage, and membranous proliferative glomerulonephritis.

Exclusion criteria: (1) Urinary infections; (2) Other acute infections (such as respiratory infections, digestive infections, etc.); (3) Patients with systemic diseases (such as allergic purpura mixed type, systemic vasculitis, Goodpasture syndrome, etc.); (4) Pathological diagnosis of DN with NDRD; (5) Patients with malignant tumors; (6) Unilateral renal atrophy; (7) Unilateral or bilateral renal artery stenosis; (8) Allergic to ultrasound contrast agents.

### Data collection

The following clinical characteristics of patients were collected: gender, age, medical history of DM, family history, body mass index (BMI), presence of hypertension, systolic blood pressure (SBP), diastolic blood pressure (DBP), mean arterial pressure, and presence of retinopathy. Laboratory parameters, including hemoglobin, serum creatinine, estimated glomerular filtration rate (eGFR, calculated by the CKD-EPI formula: Scr ≤ 0.9 ml/dl:eGFR = 144 × (Scr/0.9)^-0.411^ × (0.993)*Age and Scr > 0.9 ml/dl:eGFR = 144 × (Scr/0.9)^-1.209^ × (0.993)*Age for male; Scr ≤ 0.7 ml/dl:eGFR = 144 × (Scr/0.7)^-0.329^ × (0.993)*Age and Scr > 0.7 ml/dl:eGFR = 144 × (Scr/0.7)^-1.209^ × (0.993)*Age for female), serum albumin, glycated hemoglobin, 24-h urine protein, presence of glomerular hematuria, and urine osmotic pressure were collected before renal biopsy.

### Renal biopsy and pathological examination

The renal biopsies were performed by an experienced physician, and all renal biopsy specimens were reviewed independently by two pathologists, who coordinate all disputed cases by thorough discussion. The criteria of DN diagnosis were: mesangial proliferation, diffuse capillary glomerulosclerosis, presence or absence of K-W nodules, diffuse thickening of the glomerular basement membrane (GBM), and exudative injuries such as fibrous cap or/and hyaline thrombi [12]. Pathological diagnosis of NDRD was based on guidelines as previously described [13].

### Three-dimensional ultrasound imaging

A Philips EPIQ 7 high-end color doppler ultrasound instrument was used, and 3D imaging was completed through EPIQ 7. Probe × 6–1, frequency 1-6 Hz, and the instrument was equipped with QLAB image processing software.

This technique has been done by doctors with 5 years working experience in ultrasonic image. The patient took lateral position during examination, the ipsilateral upper limb was placed over the head. The probe was placed on the waist to get a clear maximum section along the long axis of the kidney; the 3D mode was activated to ensure that the whole kidney was included in the image. During image acquisition, patients were asked to hold their breath. The volume data of kidneys were collected, and the DICOM images were stored in the machine. Following the same method, the volume data of each kidney was acquired for three times. All images post-processing were completed by the professional and technical personnel.

**Fig. 1:**
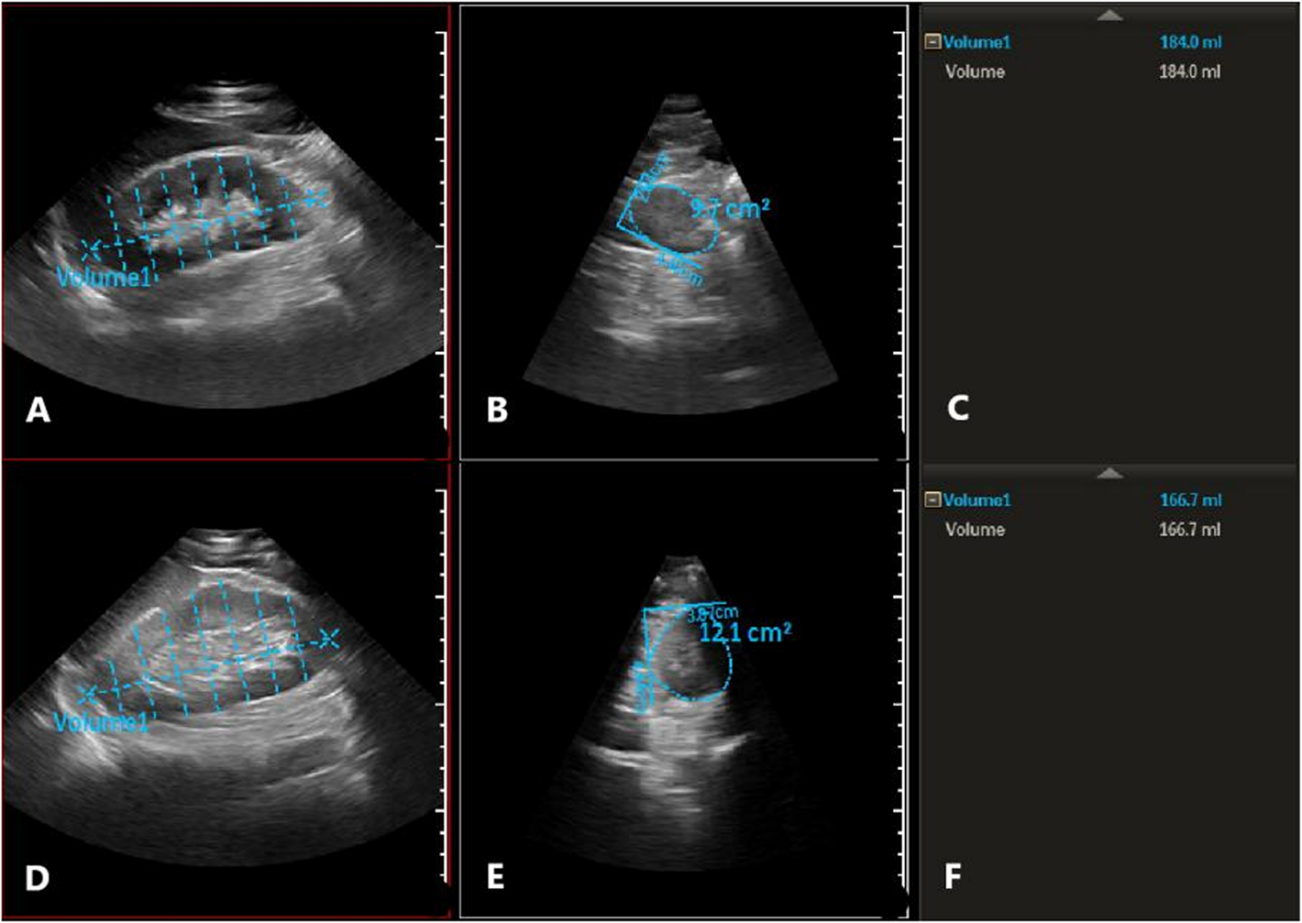
Right kidney volume measured by 3D mode. **a**, **b** and **c** showed the right kidney volume of A 58-year-old man with DN and body mass index of 24.5 in CKD garde 1. **d**, **e** and **f** showed the right kidney volume of a 61-year-old man of NDRD with body mass index of 24.7 in CKD grade 1

3D mode was selected and activated in QLAB software. Kidney volume was measured in advanced function menu. Seven layers of cross-sections were selected to estimate the volume. After determining the length of the kidney, the software automatically calculated the volume of the kidney after manually recording 7 layers (Fig. 1). The average number of the three kidney volume data was taken as the volume of the kidney.

Calculation of body surface area and kidney volume index: height and body mass were measured for all subjects using the following formula: body surface area = 0.0061 × height + 0.0128 × body mass - 0.1529, and kidney volume index = kidney volume / body surface area.

### Contrast ultrasound examination

Siemens S2000 high-end color Doppler ultrasound, probe C6–2, frequency 2-6 Hz. The instrument was equipped with Contrast Dynamics, a post-processing software for contrast ultrasound.

The patient took lateral position during examination and the ipsilateral upper limb was placed over the head. The probe was placed on the waist to get a clear maximum section along the long axis of the kidney. In this section, the parenchyma of the kidney should be parallel to the skin to minimize the sampling error caused by the position change of kidney affected by breathing. Then we started the contrast mode of the machine, activated double display, adjusted the focus position, automatically optimized the images, adjusted the settings of instrument (MI = 0.05–0.06), and reduced background gain noise. After fixing the position of the probe, the contrast agent at a dose of 0.7 ml/time was injected into the superficial vein of elbow, followed by an immediate 5 ml saline injection. In order to avoid the differences caused by different speed of injection, the time of injecting contrast agent and saline was controlled within 3 s. From the beginning of injection, the video DICOM images were stored dynamically for 3 min. During the period of corticomedullary enhancement, the patients were instructed to hold their breath, and then breathe slowly and quietly to avoid heave breathing. The kidney on the other side was examined by the same method, and the interval time between the two kidney examinations was more than 20 min.

**Fig. 2:**
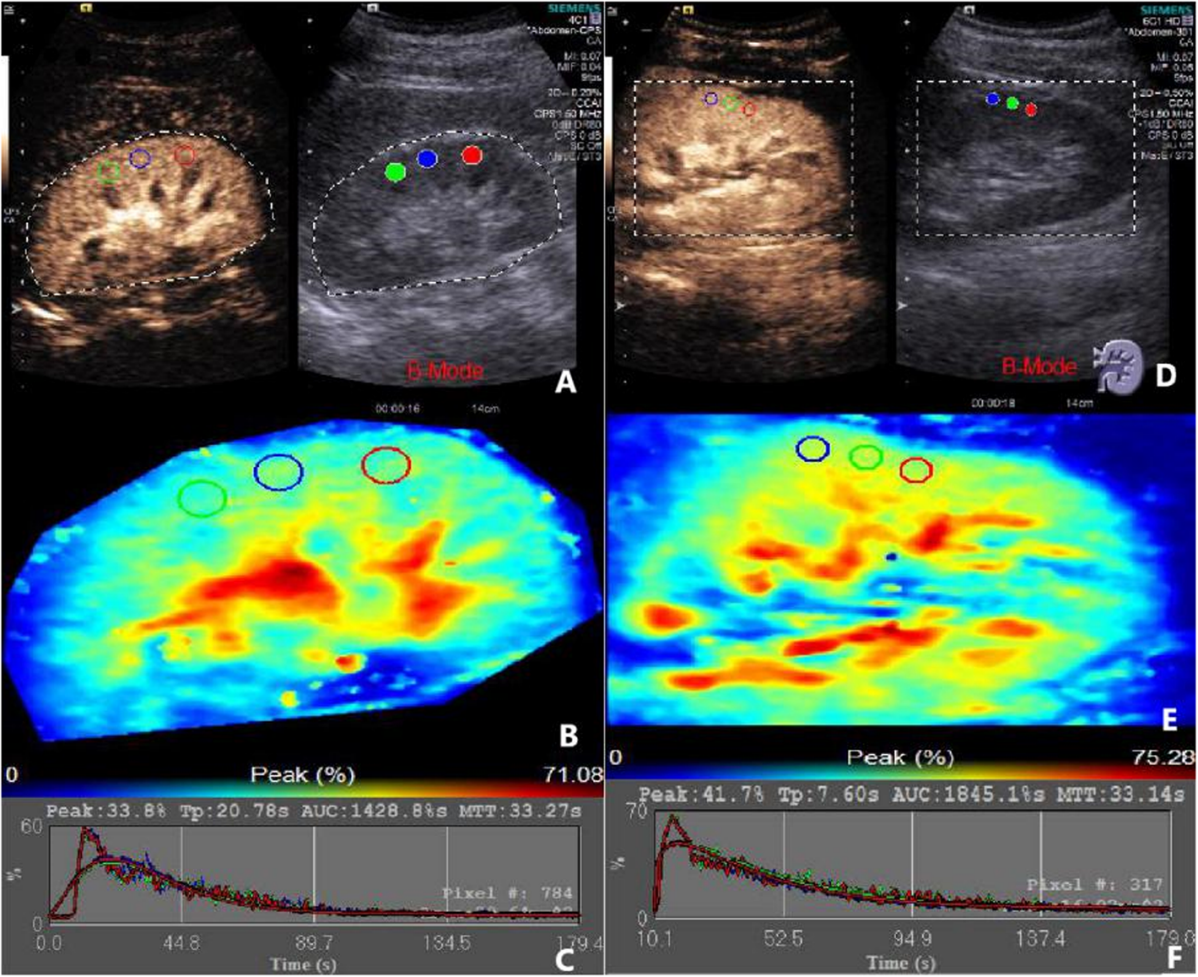
Left renal angiography by contrast ultrasound. **a**, **b** and **c** showed the image of left renal angiography of a 58-year-old man with DN and body mass index of 24.5 in stage CKD grade 1. **d**, **e** and **f** showed the image of left renal angiography of a 61-year-old man of NDRD with body mass index of 24.7 in CKD grade 1

After selecting the image, we turned on Contrast Dynamics software, selected the region of interest (ROI) in the renal cortex, and kept the ROI depth of all patients consistent when sampling. Aftering turning on the image motion compensation, the time-intensity curve was automatically generated by the software. Quantitative parameters were obtained by fitting curve: peak intensity (Peak, %), peak time (Tp). Area under curve (AUC % s), mean transit time (MTT s). We duplicated the size of the sample and measured it three times in the cortex of the kidney (Fig. 2).

Since manual selection could lead to slight inconsistence in the area size of ROI each time, we used AUC per unit area for correction. Per unit area under the curve was represented by area under curve (AUC % s)/sampling area. Slope was calculated by peak intensity (Peak, %) and peak time (Tp).

### Statistical analysis

The measurement variables were expressed as mean ± standard deviation (SD) or median (IQR). Student’s t-test was used if the results of two groups were in a normal distribution; if not, Wilcoxon two-sample test would be used. The three groups were compared with the normal distribution by using one-way ANOVA. For the results which did not fit the normal distribution, Kruskal-Wallis test was used. And SNK (Student-Newman-Keuls) method was used for posterior comparisons of two groups. The categorical variables were expressed in terms of frequency (%), and the comparison between groups used the Chi-square test or the Fisher exact probability method. This study compared diabetic nephropathy (DN) with non-diabetic renal disease (NDRD) and expected to find statistically significant differences between the two groups. Since the CKD grade with stage between the DN and NDRD groups was significantly different, further screening analysis was used in order to exclude the interference of CKD grade with stage. The used methods were CKD grade group comparison, correlation analysis, and logistic multi-factor regression. The last remaining indicators were fitted with logistic model and comprehensive predictive index. The ROC and AUC of each index and logistic regression model were plotted and calculated. When the Youden index was the largest, the cutoff value of the ROC was taken. Statistical analysis was performed by using the SAS 9.2 software, both using two-sided test and *P* < 0.05 was considered to be significant.

## Results

### General characteristics

**Fig. 3:**
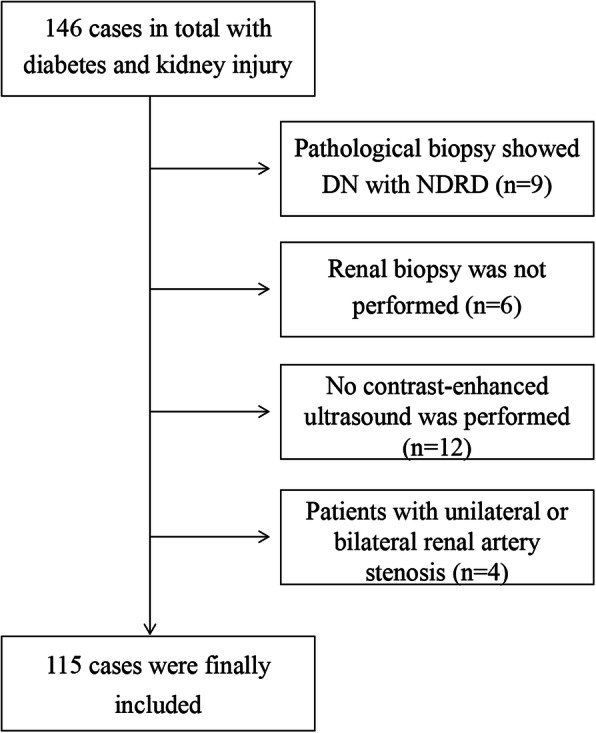
Flow chart of the study

One hundred forty-six cases in total with diabetes and kidney injury were preliminary included, and 31 cases were excluded due to the following reasons: pathological biopsy indicating the comorbidity of DN and NDRD (*n* = 9); renal biopsy was not performed (*n* = 6); no contrast-enhanced ultrasound was performed (*n* = 12); patients with unilateral or bilateral renal artery stenosis (*n* = 4). Thus, a total of 115 patients (Fig. 3) met the screening criteria, including 64 patients in the DN group and 51 patients in the NDRD group, with an age range of 24–78 years old. There were no significant differences between the DN group and the NDRD group in age distribution, gender distribution, body surface area and DBP (*p* > 0.05). The proportion of patients with history of diabetes and SBP in the DN group were higher than those in the NDRD group (*p* < 0.05), but the BMI was lower. In the NDRD group (*p* = 0.010), the proportion of hematuria in the DN group was lower than that in the NDRD group (31.25 vs. 50.98%, *p* = 0.032). Among the included samples, the DN group and the NDRD group had significant differences in the CKD grade with stage, and the proportion of CKD stage in the DN group was higher than that in the NDRD group. The results were shown in Table 1.

**Table 1:** General characteristics in DN and NDRD patients

Variable	Variable level	DN, *n* = 64	NDRD, *n* = 51	*P* value
Sex	Female	12 (18.75)	14 (27.45)	0.268
	Male	52 (81.25)	37 (72.55)	
CKD staging	1	7 (10.94)	15 (29.41)	0.001
	2	7 (10.94)	14 (27.45)	
	3	22 (34.38)	16 (31.37)	
	4	19 (29.69)	5 (9.80)	
	5	9 (14.06)	1 (1.96)	
Hematuria	No	44 (68.75)	25 (49.02)	0.032
	Yes	20 (31.25)	26 (50.98)	
Age(y)	Mean ± SD.	52.33 ± 9.71	51.94 ± 11.27	0.844
History of diabetes(y)	Median (IRQ)	12 (7,17)	4 (2,7)	< 0.001
Hypertension	No	27 (42.2)	20 (39.3)	0.828
	Yes	37 (57.8)	31 (60.7)	
Treatment for diabetes	No	7 (10.94)	16 (31.37)	0.006
	Yes	57 (89.06)	35 (68.63)	
Diabetic retinopathy	No	27 (51.92)	34 (80.95)	0.003
	Yes	25 (48.08)	8 (19.05)	
BMI (Kg/m^2^)	Mean ± SD.	25.96 ± 3.21	28.32 ± 5.43	0.010
Body Surface area(m^2^)	Mean ± SD.	1.84 ± 0.19	1.87 ± 0.23	0.462
SBP (mmHg)	Mean ± SD.	152.91 ± 21.84	135.59 ± 19.21	< 0.001
DBP (mmHg)	Mean ± SD.	86.81 ± 12.21	83.86 ± 13.47	0.187

### Biochemical indicators

There were no significant differences between the DN group and the NDRD group in urinary creatinine and serum glucose (*P* > 0.05). The outcomes of urinary protein, plasma urea, and serum creatinine were higher in the DN group than in the NDRD group (*P* < 0.05), but the glomerular filtration rate in the DN group was lower than that in the NDRD group (*P* < 0.05). The results were shown in Table 2.

**Table 2:** Biochemical indicators in DN and NDRD patients

Variable	Variable level	DN, *n* = 64	NDRD, *n* = 51	*P* value
Urinary protein(g/L)	Median (IRQ)	2.11 (1.05,2.94)	1.43 (0.46,2.4)	*0.007*
Glomerular filtration rate (ml/min)	Median (IRQ)	34.18 (21.9,55.63)	69.07 (46.11,94.28)	< 0.001
Plasma urea nitrogen (mmol/L)	Median (IRQ)	10.19 (7.22,12.95)	6.67 (5.1,7.96)	< 0.001
Serum creatinine (umol/L)	Median (IRQ)	169.55 (115.45,245.7)	97.9 (75.5142.7)	< 0.001
Urinary creatinine (mmol/L)	Median (IRQ)	4.55 (3.8,6.2)	5.4 (4,7.3)	0.076
Serum glucose (mmol/L)	Median (IRQ)	5.86 (4.32,7.55)	5.86 (4.9,6.8)	0.846
Fasting Plasma Glucose (mg/dL)	Median (IRQ)	140.4 (112, 171.8)	140.1 (108, 184)	0.920
HbA1c(%)	Median (IRQ)	7.57 (6.17, 8.97)	7.63 (6.31, 8.95)	0.710

### 3DUS and CEUS examination

There were no significant differences in right kidney volume, left kidney volume, left kidney volume index between DN group and NDRD group through the 3DUS examination (*P* > 0.05). Nevertheless, the right kidney volume index of the DN group was higher than that of the NDRD group (*P* = 0.021). The results were shown in Table 3. CEUS results revealed that there were no significant differences in terms of LK Tp, LK ROC curve rising branch slope, RK PEAK, RK Tp, RK ROC curve rising branch slope, RK AUC, RK MTT and RK Per unit area under the curve between the DN group and the NDRD group (*P* > 0.05). The LK PEAK, LK AUC, LK MTT, and LK Per unit area under the curve in the DN group were lower than those in the NDRD group, and the difference was statistically significant (*p* < 0.05). Other indicators of CEUS in both groups were similar, and the results were shown in Table 4.

**Table 3:** The results of 3DUS examination in DN and NDRD patients

Variable	Variable level	DN, *n* = 64	NDRD, *n* = 51	*P* value
Right kidney volume (ml)	Mean ± SD	180.43 ± 42.57	162.16 ± 50.29	0.073
Right kidney volume index (ml/m^2^)	Mean ± SD	98.28 ± 20.77	86.79 ± 24.39	0.021
Left kidney volume (ml)	Mean ± SD	188.58 ± 50.11	185.87 ± 51.89	0.809
Left kidney volume index (ml/m^2^)	Mean ± SD	102.88 ± 25.52	100.04 ± 27.03	0.622

**Table 4:** The results of CEUS in DN and NDRD patients

Variable	Variable level	DN, *n* = 64	NDRD, *n* = 51	*P* value
LK PEAK	Mean ± sd.	26.45 ± 8.81	31.42 ± 7.27	0.003
LK Tp	Median (IRQ)	11.59 (8.95,15.15)	12.31 (9.56, 18.09)	0.309
LK ROC curve rising branch slope	Median (IRQ)	2.15 (1.44,3.13)	2.53 (1.53, 3.47)	0.378
LK AUC	Median (IRQ)	1097.35 (845.13,2055)	1851.37 (1375.67, 2619.03)	0.007
LK MTT	Mean ± sd.	41.05 ± 17.92	47.64 ± 17.95	0.022
LK Per unit area under the curve	Median (IRQ)	53.07 (34.01,88.16)	85.73 (51.01, 116.92)	0.016
RK PEAK	Mean ± sd.	27.45 ± 7.94	30.27 ± 7.98	0.076
RK Tp	Median (IRQ)	13.3 (9.81,17.2)	11.93 (9.32,17.5)	0.789
RK ROC curve rising branch slope	Median (IRQ)	2.13 (1.54,2.52)	2.31 (1.89, 3.11)	0.095
RK AUC	Median (IRQ)	1457.65 (904.72,2078.49)	1779.97 (1330.53, 2395.43)	0.058
RK MTT	Median (IRQ)	41.7 (27.51,53.62)	44.18 (35.46, 56.74)	0.200
RK Per unit area under the curve	Median (IRQ)	57.64 (35.75,101.46)	70.63 (47.94, 123.01)	0.091

### Diagnostic performances

Since the DN and NDRD patients included in this study have significant differences in the grade of nephropathy, we further analyzed the above two groups of indicators to exclude influences from kidney disease, in order to screen out the differential indicators that interfered with the grade of nephropathy. By comparing the CKD grade with stage group, we found that there were no significant differences in hematuria, BMI, right kidney volume index and LK MTT among different patients with CKD grade with stage (*p* > 0.05, the results were shown in Supplementary Table 1). According to Spearman rank correlation analysis, CKD grade with stage has a negative correlation with LK MTT (*r* = − 0.218, *P* = 0.029, the results were shown in Supplementary Table 2). After logistic multivariate regression analysis, we found that there were no significant differences in hematuria and LK MTT between DN and NDRD patients after correction to CKD grade with stage (*p* < 0.05, the results were shown in Supplementary Table 3). Therefore, BMI and right kidney volume index were finally retained to identify whether patients are with DN or with NDRD.

**Fig. 4:**
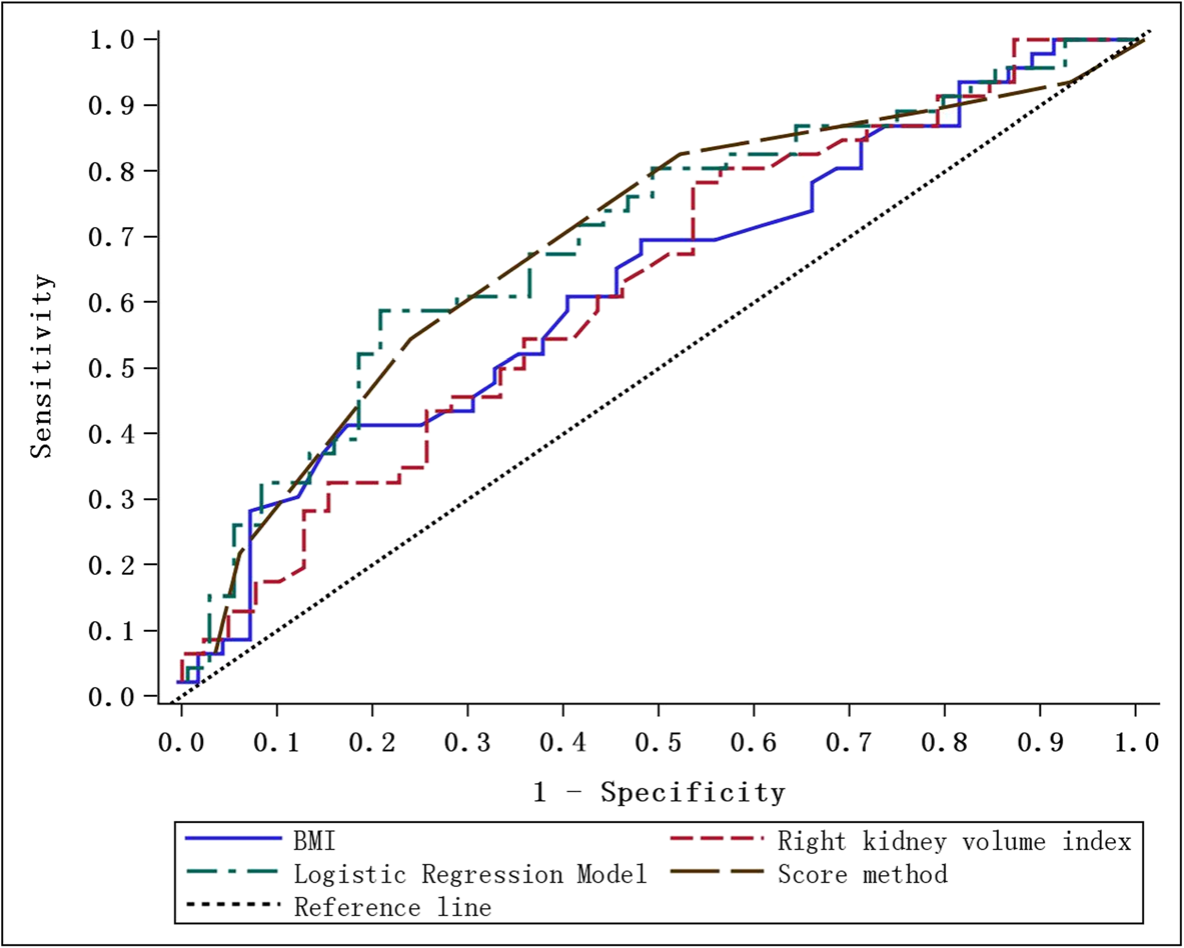
Identify the ROC curve of DN and NDRD in diabetic patients

The ROC curves of DN and NDRD identified by BMI and right kidney volume index were shown in Fig. 4. The area under the ROC curve (AUC) and the reference curve (0.5) were statistically significant (*p* < 0.05, see Table 5). The diagnostic value of both BMI and right kidney volume index was not high. Therefore, the ROC of the logistic regression model including BMI and right kidney volume index was established (Fig. 4). The ROC of the logistic regression model with an AUC of 0.703 (95% CI: 0.591–0.815) was higher than the single indicator AUC. Considering that the logistic regression model was inconvenient for clinical use, we established a new method based on BMI and right kidney volume index. The scoring method was shown in Table 6. The AUC of the score method was 0.701 (95% CI: 0.591–0.811), and the difference in AUC from the logistic regression model was not statistically significant (*p* = 0.928).

**Table 5:** ROC area (AUC)

ROC model	AUC	95% CI of AUC		Cutoff value	Sensitivity	Specificity
		Lower limit	Upper limit			
BMI	0.625 a	0.505	0.744	24.5	0.863	0.391
Right kidney volume index	0.626 a	0.506	0.746	84.5	0.783	0.462
Logistic Regression Model	0.703 a	0.591	0.815	–	–	–
Score method	0.701 ab	0.591	0.811	4.5	0.826	0.487

Remarks: a. Comparison of AUC and reference curve (0.05) *P* < 0.05; b. Comparison of AUC and Logistic Regression Model, *p* = 0.928

**Table 6:** Assignment method of score method

Index	Level	Assignment
BMI	0 < BMI < 24.5	4
	24.5 ≤ BMI < 26.5	3
	26.5 ≤ BMI < 28.5	2
	28. ≤ BMI	1
Right kidney volume index (RKVI)	0 < RKVI< 80	1
	80 ≤ RKVI< 95	2
	95 ≤ RKVI< 110	3
	110 ≤ RKVI	4
Score	Total score	Sum of BMI and RKVI

## Discussion

The prevalence of DN in T2DM patients varies from place to place [14], which depends on the selection criteria, threshold of biopsy, and population studied [15]. The current results showed that the DN incidence was 56.65% among 115 type 2 DM patients who underwent renal biopsy. Compared with NDRD patients, the DN group had significant longer disease history with diabetes, higher urinary protein and most of the patients were in the grade with stage of CKD, as well as lower glomerular filtration rate. It is accepted that the development of DN is highly related to the duration of DM, and the risk of nephropathy increases with prolonged duration of DM. Our finding was consistent with previous studies [16, 17], which showed that DN patients had a longer diabetes history. However, the diagnostic performance of this clinical feature for DN is still controversial. Clinically, microalbuminuria is an important index to assess the presence of renal dysfunction and progression of DN [18]. Just as our study found, urinary protein in DN group was significantly higher than that of the NDRD group. However, it is not accurate to evaluate the severity or prognosis simply based on the degree of proteinuria. It is now well recognized that not all diabetic patients who develop renal function failure have massive albuminuria [19, 20]. In a cohort of 6072 individuals, only 17% of patients with type 2 diabetes with a mean duration of 8 years and an eGFR < 60 ml/min per 1.73 m^2^ had normoalbuminuria [20]. Progressive stages of glomerular hyperfiltration and a decline in the GFR are also the classic description of DN [21]. From the study, it revealed that later grade with stage of CKD and lower glomerular filtration rate were found worse in DN group with significance. Yet, in recent years, this concept has been increasingly challenged as evidence from the past years has witnessed a more heterogeneous manifestation of DN. From the above, none of these indicators could be applied alone for efficient and accurate diagnosis of DN, and therefore we combined ultrasound technology to achieve higher value and possibilities for the diagnosis.

The diagnosis of renal disease is commonly based on GFR value, urinary abnormalities such as proteinuria and hematuria, and ultrasound structural kidney alterations. Ultrasound (US) with color doppler (CD) imaging [22] is the first imaging technique to be performed when CKD is suspected or diagnosed [23]. In CKD, longitudinal kidney diameter [24], parenchymal thickness/echogenicity, and urinary tract conditions should be evaluated [25]. Longitudinal renal diameter is considered a pivotal marker of CKD since it decreases as GFR progressively declines [26]. In addition, some studies [27] have demonstrated that kidney volume is a more precise parameter of kidney function [28]. By using two-dimensional ultrasound measurement, information such as kidney size and shape, renal capsular, internal echo and the thickness of cortex and medulla could be obtained. In three dimensions mode, the volume probe automatically performed a three-dimensional scan of the kidney. The software was used to calculate the renal volume, body surface area and renal volume index so as to provide more accurate morphological structure of kidney. In this study, we found that the right kidney volume index was higher than that of the NDRD group, which is consistent with the conclusions of previous researches.

CEUS has been widely accepted [29] and used in the perfusion studies on liver, cardiac muscles, brain and even musculoskeletal perfusion of lower extremities [30]. It is an excellent imaging approach associated with low mechanical index (MI) US and microbubble-based contrast agents [31]. Since the contrast agent has no nephrotoxicity, the application of CEUS for the detection of renal dysfunction is considered to be much safer [32]. CEUS has been used to assess early kidney dysfunction [33] and changes of renal perfusion [34] in diabetic nephropathies [35, 36]. Renal microcirculation perfusion could be analyzed by CEUS, and the situation of DN renal microperfusion in different stages could also be evaluated, so as to determine the situation of renal damage and provide quantitative indexes reflecting renal blood perfusion for DN. We found that the LK PEAK, LK AUC, LK MTT, and LK Per unit area under the curve in the DN group were lower in this study. However, we did not find any significant differences on the CEUS quantitative index in the final analysis of regression, which probably resulted from the small sample size or the study design or inclusion and exclusion criteria.

Hence, we established a logistic regression model, in which BMI and right kidney volume index were included eventually. The AUC of the both indicators and the reference curve were statistically significant. For easy clinical use, we thus built a new method based on BMI and right kidney volume index. With respect to the significant increase in BMI in patients with DN compared with NDRD, the possible reason is that DN patients often have poor blood glucose control and abnormal glucose metabolism, leading to abnormal lipid metabolism and overweight.

To our knowledge, this is the first study on the diagnostic value of 3DUS and CEUS on diabetic patients with kidney injury. However, there are still some limitations in the current study. First, the CKD grade with stage of patients in DN and NDRD groups is inconsistent, which means the severity of renal injury in DN group is relatively higher. This could possibly result in some bias in 3DUS and CEUS presentation. Additionally, the total sample size is small or study design issue, and therefore, a larger population with well designed study is needed in the future.

## Conclusion

3DUS might be used to evaluate the severity of renal pathology and may be potentially valuable for auxiliary diagnosis in DN patients.

## Supplementary information

**Additional file 1: Supplement Table 1.** Comparison of different CKD staging Groups. **Supplement Table 2.** Correlation analysis between CKD staging and BMI, Right kidney volume index and LK MTT. **Supplement Table 3.** Comparison of BMI, Hematuria, Right kidney volume index, LK MTT in DN and NDRD patients after correcting CKD staging.

## Data Availability

All data are available by request of our corresponding author.

## Abbreviations

DM: Diabetes Mellitus
DN: Diabetic Nephropathy
NDRD: Non-Diabetic Renal Diseases
3DUS: Three-Dimensional Ultrasound
CEUS: Contrast Enhanced Ultrasound
TIC: Time-intensity curve
KV: Kidney Volume
KVI: Kidney Volume Index
Peak: Peak intensity
Tp: Time To Peak
RBSROC: Curve rising branch slope
MTT: Mean Transit Time
AUC: Area Under the Curve
AUCPUA: Area Under the Curve Per Unit Area
ROC: Receiver Operating Characteristic Curves
BMI: Body Mass Index
BSA: Body Surface Area
SBP: Systolic Blood Pressure
DBP: Diastolic blood pressure
UP: Urine Protein
GFR: Glomerular Filtration Rate
PUN: Plasma Urea Nitrogen
SC: Serum Creatinine
UC: Urinary Creatinine
GLU: Serum Glucose
HIS: History of diabetes
DR: Diabetic Retinopathy
HEM: Hematuria
