# Supplementary material for: Potential value of three-dimensional ultrasonography in diagnosis of diabetic nephropathy in Chinese diabetic population with kidney injury
Source: BMC Nephrol. 2020 Jun 29;21:243. doi: 10.1186/s12882-020-01902-w (PMC7325142; doi:10.1186/s12882-020-01902-w)
Supplement: Supplementary file 1 — Additional file 1: Supplement Table 1. Comparison of different CKD staging Groups. Supplement Table 2. Correlation analysis between CKD staging and BMI, Right kidney volume index and LK MTT. Supplement Table 3. Comparison of BMI, Hematuria, Right kidney volume index, LK MTT in DN and NDRD patients after correcting CKD staging. [file 12882_2020_1902_MOESM1_ESM.docx]

**Supplement Table 1**. Comparison of different CKD staging Groups

| Variable | Variable level | Grade 1, n=22 | Grade 2-3, n=59 | Grade 4-5, n=34 | p |
| --- | --- | --- | --- | --- | --- |
| Hematuria | No | 9 (40.91) | 36 (61.02) | 24 (70.59) | 0.084 |
|  | Yes | 13 (59.09) | 23 (38.98) | 10 (29.41) |  |
| History of diabetes(y) | Median (IRQ) | 3 (2,7) | 8 (5,13) | 10.5 (7,15) | 0.002 |
| BMI(Kg/m2) | Mean ± sd. | 28.5 ± 6.59 | 26.78 ± 4.29 | 26.43 ± 2.7 | 0.776 |
| SBP(mmHg) | Mean ± sd. | 136.23 ± 21.86 | 141.58 ± 22.22 | 157.38 ± 18.06 | <0.001 |
| Urinary protein(g/L) | Median (IRQ) | 1.12 (0.51,2.06) | 1.47 (0.77,2.56) | 2.54 (1.98,3.33) | <0.001 |
| Glomerular filtration rate(ml/min) | Median (IRQ) | 98.35 (95.58,108.99) | 50.86 (38.74,68.97) | 21.05 (14.99,25.5) | <0.001 |
| Plasma urea nitrogen(mmol/L) | Median (IRQ) | 5.5 (4.26,6.98) | 7.49 (6.13,9.14) | 13.12 (11.43,15.96) | <0.001 |
| Serum creatinine(umol/L) | Median (IRQ) | 71.7 (55.6,76.2) | 118 (94,144.3) | 253.7 (217.3,314.8) | <0.001 |
| Right kidney volume index(ml/m2) | Mean ± SD. | 101.74 ± 16.45 | 88.28 ± 24.42 | 95.25 ± 23.59 | 0.090 |
| LK PEAK | Mean ± SD. | 32.81 ± 7.45 | 28.53 ± 8.46 | 25.9 ± 8.24 | 0.015 |
| LK AUC | Median (IRQ) | 1957.37 (1257.53,2935.4) | 1668.9 (902.17,2338.05) | 1119.5 (837.17,2076.9) | 0.037 |
| LK MTT | Mean ± SD. | 48.11 ± 18.43 | 44.48 ± 18.12 | 40.16 ± 17.86 | 0.256 |
| LK Per unit area under the curve | Median (IRQ) | 65.06 (49.73,101.55) | 75.83 (38.76,128.42) | 50.16 (30.49,73.14) | 0.026 |

**Supplement Table 2**. Correlation analysis between CKD staging and BMI, Right kidney volume index and LK MTT

| Variable | Spearman Correlation | CKD staging | BMI | Right kidney volume index(ml/m1) | LK MTT |
| --- | --- | --- | --- | --- | --- |
| BMI | r | 0.028 | - | 0.072 | 0.089 |
|  | *P* | 0.767 | - | 0.512 | 0.374 |
| Right kidney volume index(ml/m1) | r | -0.093 | 0.072 | - | -0.168 |
|  | *P* | 0.395 | 0.512 | - | 0.147 |
| LK MTT | r | -0.218 | 0.089 | -0.168 | - |
|  | *P* | 0.029 | 0.374 | 0.147 | - |

**Supplement Table 3**. Comparison of BMI, Hematuria, Right kidney volume index, LK MTT in DN and NDRD patients after correcting CKD staging

| Model | Independent variable | Degrees of freedom | | Wald Chi-square | P  value |
| --- | --- | --- | --- | --- | --- |
| Logistic  regression models | BMI(Kg/m2) | | 1 | 7.856 | 0.005 |
|  | CKD staging | | 4 | 17.94 | 0.001 |
| Logistic  regression models | Hematuria | | 1 | 2.354 | 0.125 |
|  | CKD staging | | 4 | 15.12 | 0.004 |
| Logistic  regression models | Right kidney volume index(ml/m2) | | 1 | 6.072 | 0.014 |
|  | CKD staging | | 4 | 9.916 | 0.042 |
| Logistic  regression models | LK MTT | | 1 | 0.990 | 0.320 |
|  | CKD staging | | 4 | 12.20 | 0.016 |
